# Awareness, attitudes, and experiences of using digital health services among patients with chronic diseases at King Abdulaziz Medical City

**DOI:** 10.3389/fdgth.2026.1887874

**Published:** 2026-09-28

**Authors:** Seham A. Elgamal, Rahmah Alalawi, Neama Y. Hantira, Rana Alsulami, Suzan Abdulsubhan, Danah Abutaleb, Batool Althobaiti, Rafa Binzagr, Shahad Almehmadi, Dareen Alqhtani

**Affiliations:** 1 College of Nursing, King Saud bin Abdulaziz University for Health Sciences, Jeddah, Saudi Arabia; 2 King Abdullah International Medical Research Center (KAIMRC), Jeddah, Saudi Arabia; 3 Ministry of the National Guard Health Affairs, Jeddah, Saudi Arabia; 4 Community Health Nursing Department, Faculty of Nursing, Alexandria University, Alexandria, Egypt

**Keywords:** attitude, awareness, chronic illness, digital health, mHealth, Saudi Arabia

## Abstract

**Background:**

Digital health technologies have transformed chronic disease management by supporting remote monitoring, personalized care, and improved communication between patients and healthcare providers. Applications such as mobile health platforms, telemedicine, and wearable devices can enhance self-management and continuity of care for individuals with chronic illnesses. In Saudi Arabia, digital health platforms, including Sehhaty, have been introduced to improve healthcare accessibility; however, patient utilization remains suboptimal.

**Aim:**

This study aimed to assess patients’ awareness, attitudes, and experiences regarding the use of digital health services among individuals living with chronic diseases.

**Methods:**

A descriptive cross-sectional study was conducted among patients with confirmed chronic illnesses attending outpatient clinics at King Abdulaziz Medical City. Data were collected using a structured questionnaire assessing sociodemographic characteristics, awareness, experience, digital health utilization patterns, and attitudes toward digital health application use. Descriptive and inferential statistical analyses were performed to identify factors associated with awareness and attitudes.

**Results:**

Nearly half of the participants (45.5%) demonstrated low digital health awareness, while 50.6% exhibited positive attitudes toward digital health applications. Sehhaty was the most commonly used application (38.9%), and 68.5% used the internet to obtain health-related information. Digital health awareness was strongly positively correlated with attitudes toward digital health applications (*r* = 0.693, *p* < 0.001). Higher awareness, use of digital health applications, internet use for health information, and younger age were significant predictors of more positive attitudes (*p* < 0.05).

**Conclusion:**

Nearly half of the patients with chronic illnesses demonstrated low digital health awareness, although attitudes toward digital health applications were generally positive. Enhancing digital literacy and integrating digital health education into routine care may improve the adoption and effective use of digital health technologies in chronic disease management.

## Introduction

Chronic diseases, including diabetes, hypertension, and heart disease, are among the leading causes of morbidity and mortality worldwide, placing substantial pressure on healthcare systems and patients (1). Management of these conditions often requires long-term treatment, continuous monitoring, and lifestyle modification (2, 3); however, many patients face barriers, including limited access to healthcare services, insufficient support systems, and low engagement in self-care practices (4).

In response to these challenges, digital health technologies have emerged as promising solutions to improve healthcare delivery and patient outcomes. Digital health encompasses a wide range of tools, including telemedicine platforms, mobile health (mHealth) applications, electronic health records (EHRs), and wearable devices, all designed to enhance accessibility, quality, and efficiency of healthcare services (5). These technologies enable patients to remotely manage their conditions, access personalized health information, and take a more active role in their healthcare management (6).

In Saudi Arabia, the Ministry of Health has introduced several digital health applications, including Mawid, Sehha, and Mawared, to facilitate healthcare services such as appointment scheduling and virtual consultations. More recently, these services were consolidated into a unified national platform, Sehhaty, which has become the primary digital health application for healthcare service delivery as of the first quarter of 2023. This integration reflects the Kingdom's ongoing commitment to advancing digital transformation and strengthening the accessibility, efficiency, and continuity of healthcare delivery through digital health systems (7).

Evidence suggests that digital health applications improve communication between patients and healthcare providers, enhance treatment adherence, and empower patients in clinical decision-making processes (8). For example, in Saudi Arabia, applications such as Seha provide real-time virtual consultations, highlighting the expanding role of digital health in chronic disease management (9).

Despite these benefits, adoption of digital health technologies remains influenced by multiple factors, including digital literacy, privacy concerns, and sociodemographic characteristics such as age, education, and socioeconomic status (10). Therefore, the study aims to assess patients’ awareness, attitudes, and experiences regarding the use of digital health services among individuals living with chronic diseases.

### Significance of the study

Digital health technologies have become increasingly important in modern healthcare systems, particularly in the management of chronic diseases, which remain a leading cause of morbidity and mortality worldwide. These technologies, including mobile health applications, telemedicine, and electronic health systems, have been shown to improve access to care, enhance self-management, and support patient engagement in long-term disease control (5). Recent evidence also highlights that digital interventions can improve health literacy and patient outcomes among individuals with chronic conditions by facilitating continuous access to health information and promoting self-care behaviors (11).

Despite the expanding use of digital health solutions, disparities in awareness, utilization, and acceptance remain significant among patients with chronic illnesses. Systematic reviews indicate that factors such as age, education level, digital literacy, and socioeconomic status strongly influence engagement with digital health tools (10, 12).

Furthermore, limited understanding of digital health features and concerns regarding usability and trust continue to hinder optimal adoption, particularly among older adults and patients with multimorbidity (13). Therefore, assessing awareness, attitudes, and utilization patterns is essential to identify gaps and barriers in digital health engagement.

In Saudi Arabia, national digital health transformation initiatives, including the integration of platforms such as Sehhaty, reflect a strong commitment to improving healthcare accessibility and service delivery (7, 9). However, empirical evidence examining patients’ awareness, attitudes, and real-world use of these applications, especially among individuals with chronic diseases, remains limited (13, 14). Understanding these factors is crucial for optimizing digital health implementation strategies, improving patient-centered care, and aligning with the objectives of Saudi Vision 2030, which emphasizes digital transformation and enhanced healthcare efficiency (15, 16).

This study therefore provides valuable insights into how patients perceive and engage with digital health services, supporting the development of targeted interventions to improve adoption and effectiveness.

### The aim of the study

The study aims to assess patients’ awareness, attitudes, and experiences regarding the use of digital health services among individuals living with chronic diseases.

### Study objectives: therefore, this study aimed to:

1. Assess the level of digital health awareness among patients with chronic diseases.
2. Assess patients’ attitudes toward digital health applications.
3. Describe patients’ experiences and utilization patterns of digital health services.
4. Examine the relationship between digital health awareness and attitudes toward digital health applications.
5. Identify factors associated with attitudes toward digital health applications among patients with chronic diseases.

### Research questions

1. What is the level of awareness among patients with chronic diseases regarding digital health services?
2. What are the patterns of digital health service utilization (experience) among patients with chronic diseases?
3. What are the attitudes of patients with chronic diseases toward digital health services?
4. What is the relationship between attitudes toward digital health services and sociodemographic characteristics among patients with chronic diseases?

## Theoretical and conceptual framework

This study was guided by the Unified Theory of Acceptance and Use of Technology (UTAUT), which provides a framework for understanding individuals’ acceptance and use of technology through key determinants such as performance expectancy, effort expectancy, social influence, and facilitating conditions (17). The model has been widely applied in digital health research to understand patients’ acceptance and use of mobile health applications and telemedicine services (10, 18, 19).

In the context of the present study, the UTAUT framework provided a theoretical basis for examining factors associated with patients’ attitudes toward digital health applications, particularly digital health awareness and prior engagement with digital health technologies. These factors were considered relevant to patients’ perceptions and acceptance of digital health applications (20). The study also described patterns of digital health service utilization to provide a broader understanding of patients’ engagement with these services.

The conceptual framework was developed based on the UTAUT model and relevant literature (17). It proposes that sociodemographic characteristics, digital health awareness, and digital engagement and experience are associated with patients’ attitudes toward digital health applications. In the present study, attitude toward digital health applications was the primary dependent variable in the inferential analysis, whereas utilization patterns were examined descriptively rather than modeled as an outcome. Accordingly, the framework does not assume a mediation or causal pathway between attitude and utilization. This approach provides a UTAUT-informed framework for examining factors associated with patients’ attitudes while describing their patterns of digital health service use (Figure 1).

**Figure 1:**
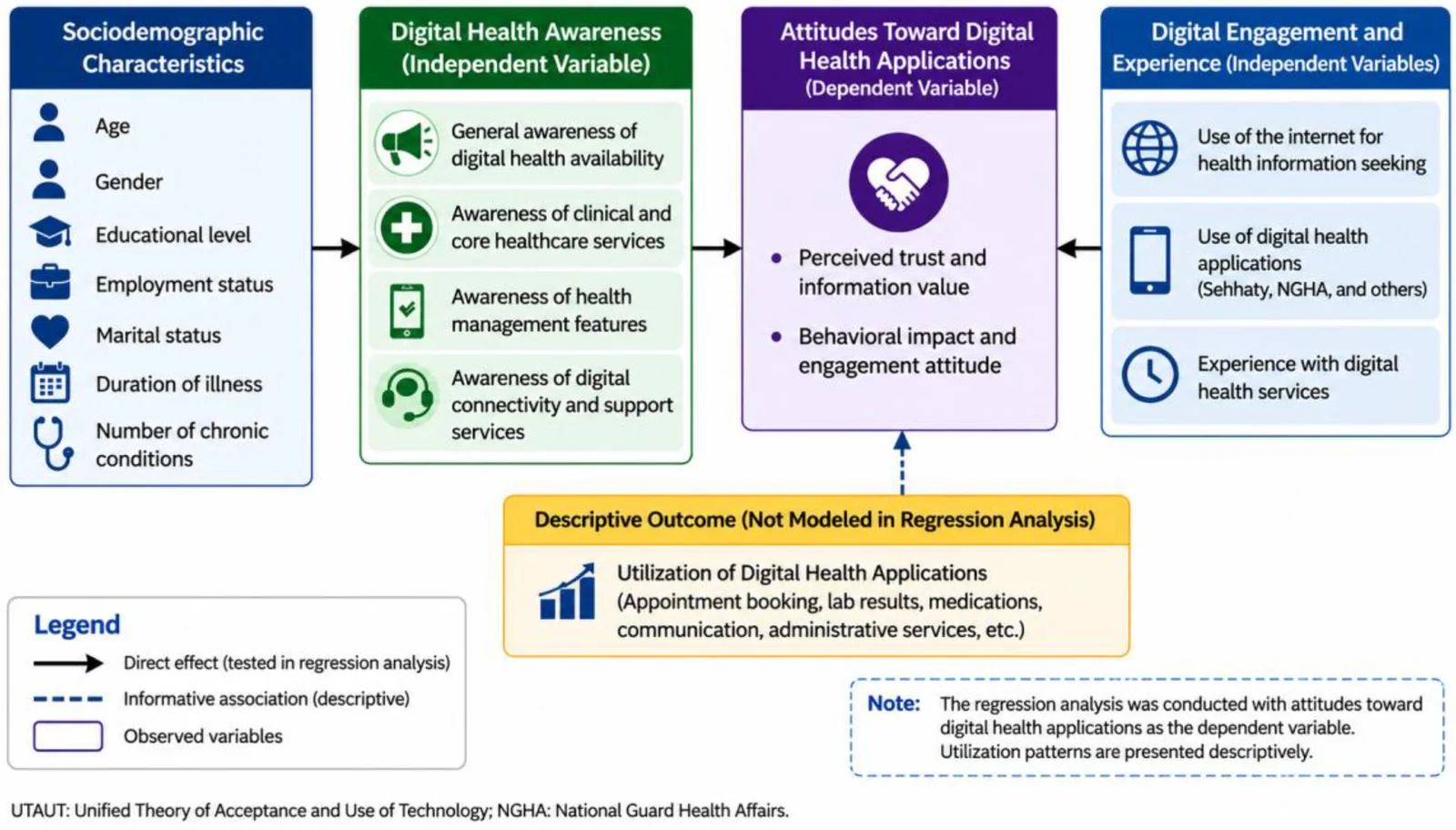
Conceptual framework of factors influencing attitudes toward and utilization of digital health applications among patients with chronic diseases. Created by the authors, 2026. The framework is based on the UTAUT model (17).

## Methodology

### Study design

A descriptive cross-sectional design was employed.

### Study settings

The study was conducted at King Abdulaziz Medical City (KAMC) in Jeddah, Saudi Arabia. King Abdulaziz Medical City (KAMC) in Jeddah, Saudi Arabia, is a tertiary healthcare institution operated by the Ministry of National Guard Health Affairs (MNGHA). It provides comprehensive specialized healthcare services, including medical, surgical, emergency, critical care, and outpatient services, and serves as a major referral center in the Western Region. KAMC is also involved in medical education and research through its affiliation with King Saud bin Abdulaziz University for Health Sciences and supports Saudi Arabia's healthcare transformation initiatives under Vision 2030 (21).

### Study subjects

The target population consisted of adult patients diagnosed with chronic diseases, including diabetes mellitus, hypertension, cardiovascular diseases, asthma, and other long-term health conditions, who were receiving treatment and follow-up care at the outpatient clinics of King Abdulaziz Medical City. Adult patients of both sexes aged 18 years and older were eligible to participate in the study. Participants were required to have a confirmed diagnosis of at least one chronic disease and the ability to understand and complete the study questionnaire independently. The only exclusion criterion was a documented diagnosis of psychiatric illness that could affect the participant's ability to provide reliable responses.

### Sample size

The required sample size was calculated using the standard formula for cross-sectional studies described by Daniel (1999) (22) (Equation 1):

$$n=\frac{Z^{2}P(1-P)}{d^{2}}$$ (1)

Where *n* represents the required sample size, *Z* is the standard normal deviation corresponding to a 95% confidence level (1.96), *P* is the estimated population proportion assumed to be 50% (0.5), and *d* is the margin of error set at 5% (0.05) (Equation 1). Based on this calculation, the minimum required sample size was 384 participants (22). A total of 433 eligible patients were approached to participate in the study. Of these, 391 agreed to participate and were included in the final analysis, yielding a response rate of 90.3%. Participant recruitment was discontinued once the required sample size had been achieved.

### Sampling technique

A non-probability convenience sampling technique was used to recruit adult patients with chronic diseases who met the inclusion criteria during the data collection period at King Abdulaziz Medical City.

### Study tools (data collection instruments)

The data collection instrument used in this study was a structured self-administered questionnaire developed following an extensive review of the relevant literature on digital health applications and chronic disease management. The questionnaire was designed to assess participants’ sociodemographic and clinical characteristics, awareness of digital health services, attitudes toward digital health applications, and patterns of digital health utilization among patients with chronic illnesses.

The questionnaire consisted of four main sections:

#### Section I: sociodemographic and clinical characteristics

This section collected participants’ demographic and clinical data, including age, gender, marital status, educational level, employment status, type and number of chronic diseases, and duration since diagnosis.

#### Section II: digital health awareness scale

This section assessed participants’ awareness of digital health applications and services. It included 19 items covering awareness of digital health availability, healthcare service delivery, health management features, and digital connectivity services. Responses were recorded as “Yes” or “No,” with higher scores indicating greater awareness of digital health tools. The total awareness score was converted into a percentage score and categorized as low (<50%), moderate (50% to <75%), and high (≥75%).

#### Section III: experience and digital health utilization patterns

This section examined participants’ self-reported actual use and experience with digital health technologies, including internet use for health-related information seeking, use of digital health applications such as Sehhaty and NGHA applications, and patterns of utilization across different digital health services. These services included navigation and access services, communication and consultation services, medication and health management services, administrative and family health services, and digital support and integration services. This section assessed participants’ actual utilization behaviors rather than their familiarity with or preferences for digital health technologies.

#### Section IV: attitude toward digital health applications scale

This section assessed participants’ attitudes toward digital health technologies across two domains: perceived trust and information value, and behavioral impact and engagement. It consisted of 16 items measured on a Likert scale, with higher scores indicating more positive attitudes toward digital health applications.

### Validity and reliability

To ensure content validity, the study instruments were reviewed by a panel of six experts in nursing education and health sciences research, who evaluated the relevance, clarity, and appropriateness of the questionnaire items. To ensure linguistic equivalence and conceptual accuracy, a forward–backward translation process was conducted from English to Arabic.

A pilot study was then carried out on 10% of the target population (*n* = 39) to assess the clarity, feasibility, and average time required for questionnaire completion. Based on the pilot testing, minor refinements were made to improve wording and comprehension. Participants included in the pilot study were excluded from the final analysis to avoid contamination of the main study sample.

The overall internal consistency of the instrument was excellent, with a Cronbach's alpha coefficient of 0.973. The reliability of the subscales was also high, with Cronbach's alpha values of 0.933 for the Digital Health Awareness Scale and 0.963 for the Attitude Toward Digital Health Applications Scale, indicating strong internal consistency and reliability. Although the overall Cronbach's alpha was very high (0.973), indicating excellent internal consistency, such high values may also reflect some degree of item redundancy. Nevertheless, the questionnaire was developed to comprehensively assess multiple dimensions of digital health awareness and attitudes, supporting its content validity.

### Data collection process

Ethical approval for this study was obtained from the Institutional Review Board of the King Abdullah International Medical Research Center (KAIMRC) (SPJ24/003/6; IRB/ 0000054724/24). In addition, official permission to conduct the study was secured from the directors of the selected study setting.

All participants were provided with detailed information regarding the study's purpose, procedures, confidentiality measures, and their rights as participants before enrollment. Written informed consent was obtained from all participants, and participation was entirely voluntary and anonymous, with the right to withdraw at any time without penalty.

Data collection was conducted from December 2024 to March 2025 by trained research assistants in a controlled and private setting to ensure participants’ comfort, privacy, and data quality. The average time required to complete the questionnaire ranged from 7 to 10 minutes.

### Data management and analysis plan

Data were coded, cleaned, and entered into the Statistical Package for the Social Sciences (SPSS) version 26 for analysis. Before analysis, data were checked for completeness, missing values, and outliers to ensure data accuracy and consistency. Descriptive statistics, including frequencies, percentages, means, and standard deviations, were used to summarize participants’ sociodemographic characteristics, clinical profiles, digital health awareness levels, utilization patterns, and attitudes toward digital health applications.

For inferential analysis, Pearson's correlation coefficient was used to examine the relationship between digital health awareness and attitudes toward digital health applications. The strength of correlation was interpreted based on established thresholds. Additionally, multiple linear regression analysis was performed to identify predictors of attitudes toward digital health applications, including age, internet use for health information seeking, digital health application use, and overall awareness score. The assumptions of regression analysis, including normality, linearity, multicollinearity, and homoscedasticity, were assessed before model testing.

A *p*-value of less than 0.05 was considered statistically significant for all analyses. Results were presented using tables and narrative summaries to provide a comprehensive interpretation of the findings.

### Ethical considerations

Ethical approval was obtained from the Institutional Review Board of the King Abdullah International Medical Research Center (KAIMRC). Ethical considerations were rigorously followed throughout the study in accordance with the principles of the Declaration of Helsinki. Participants were provided with detailed information about the study objectives and procedures and were asked to provide written informed consent before participation. Confidentiality and anonymity were strictly maintained, and participants were informed of their right to withdraw from the study at any time without any penalty or impact on their medical care. The study was conducted in accordance with international ethical standards for research involving human subjects, ensuring respect for participants’ autonomy, beneficence, and justice.

## Results

Table 1 presents the sociodemographic characteristics of the study participants (*n* = 391). More than half of the participants were female (59.3%), while males represented 40.7% of the sample. Participants’ ages ranged widely, with a mean age of 49.8 ± 15.9 years. The largest age group was 50–59 years (24.0%), followed by 40–49 years (20.7%), indicating that most participants were middle-aged or older adults, which is expected among individuals living with chronic illnesses.

**Table 1:** Sociodemographic characteristics of the study participants (*n* = 391).

Study participant characteristics	*n* (391)	%
Gender		
Female	232	59.3
Male	159	40.7
Age (years)		
18–29	54	13.8
30–39	48	12.3
40–49	81	20.7
50–59	94	24
60–69	68	17.4
70 and older	46	11.8
Mean ± SD	49.8 ± 15.9 year	
Educational level		
Illiterate	34	8.7
Primary school	49	12.5
Middle school	43	11
High school	93	23.8
Diploma	24	6.1
Bachelor's degree	134	34.3
Higher education	14	3.6
Employment status		
Employed	127	32.5
Homemaker	125	32
Retired	73	18.7
Student	42	10.7
Unemployed	19	4.9
Self-employed	5	1.3
Marital status		
Single	59	15.1
Married	262	67
Divorced	25	6.4
Widowed	45	11.5

Regarding educational level, approximately one-third of the participants held a bachelor's degree (34.3%), while 23.8% had completed high school education. However, a noticeable proportion had low educational attainment, including illiterate participants (8.7%) and those with only primary education (12.5%). These findings suggest variability in educational background, which may influence digital health literacy and technology use.

In terms of employment status, employed participants (32.5%) and homemakers (32.0%) constituted the largest groups, followed by retired individuals (18.7%). This distribution reflects the diverse socioeconomic backgrounds of the participants and may influence access to and engagement with digital health technologies. With respect to marital status, most participants were married (67.0%), whereas smaller proportions were single (15.1%), widowed (11.5%), or divorced (6.4%).

Overall, the demographic profile indicates a predominantly middle-aged, married population with varied educational and occupational backgrounds, factors that may play an important role in shaping awareness, attitudes, and usability of digital health applications.

Table 2 illustrates the chronic disease profile and duration since diagnosis among the study participants (*n* = 391). Most participants (73.9%) reported having one chronic condition, while 18.9% had two chronic conditions and 7.2% experienced three or more conditions, indicating the presence of multimorbidity among a considerable proportion of the sample.

**Table 2:** Chronic disease profile of the study participants (*n* = 391).

Chronic disease profile	*n* (391)	%
Number of chronic conditions		
One condition	289	73.9
Two conditions	74	18.9
Three or more conditions	28	7.2
Single chronic condition		
Diabetes mellitus	89	22.8
Hypertension	70	17.9
Cardiovascular diseases	24	6.1
Respiratory diseases	37	9.5
Thyroid diseases	11	2.8
Other chronic conditions	58	14.8
Multiple chronic conditions (comorbidities)		
Diabetes mellitus and hypertension	63	16.1
Diabetes mellitus, hypertension, and cardiovascular diseases	28	7.2
Diabetes mellitus and cardiovascular diseases	5	1.3
Hypertension + cardiovascular diseases	6	1.5
Duration since the initial diagnosis		
Less than 1 year	27	6.9
1 to 9 years	157	40.2
10 to 19 years	108	27.6
20 to 29 years	55	14.1
30 to 39 years	29	7.4
More than 40 years	15	3.8

Among participants with a single chronic illness, diabetes mellitus was the most reported condition (22.8%), followed by hypertension (17.9%) and respiratory diseases (9.5%). Cardiovascular diseases accounted for 6.1% of cases, whereas thyroid diseases were the least commonly reported condition (2.8%). In addition, 14.8% of participants reported other chronic conditions.

Regarding comorbidities, diabetes mellitus combined with hypertension was the most prevalent combination (16.1%), followed by diabetes mellitus, hypertension, and cardiovascular diseases (7.2%). Smaller proportions of participants reported diabetes mellitus with cardiovascular diseases (1.3%) or hypertension with cardiovascular diseases (1.5%). These findings highlight the substantial burden of multiple chronic conditions among the study population, which may increase the need for continuous monitoring and digital health support.

Concerning the duration since the initial diagnosis, the largest proportion of participants (40.2%) had been diagnosed for 1–9 years, followed by 27.6% who had lived with their condition for 10–19 years. Smaller proportions reported disease durations of 20–29 years (14.1%), 30–39 years (7.4%), and more than 40 years (3.8%). Only 6.9% had been diagnosed within the past year.

Overall, the findings suggest that most participants were living with long-term chronic illnesses, emphasizing the importance of sustainable and accessible digital health interventions for ongoing disease management.

Table 3 presents the mean percentage scores of digital health awareness across four domains, as well as the overall awareness score among the study participants. The findings showed that participants demonstrated the highest level of awareness in the domain of general awareness of digital health availability (Mean = 80.35%, SD = 30.23), indicating a relatively strong awareness of the existence of digital health services.

**Table 3:** Mean percentage scores of digital health awareness across domains.

Digital health awareness domain	Mean (%)	SD
General awareness of digital health availability	80.35	30.23
Awareness of clinical and core healthcare services	58.49	35.11
Awareness of health management features	35.40	35.57
Awareness of digital connectivity and support services	37.02	37.46
Total digital health awareness score	51.35	30.69
Digital health awareness categories	No.	%
Low awareness (<50%)	178	45.5
Moderate awareness (50 < 75%)	116	29.7
High awareness (≥75%)	97	24.8

In contrast, lower levels of awareness were observed in the domains of health management features (Mean = 35.40%, SD = 35.57) and digital connectivity and support services (Mean = 37.02%, SD = 37.46), reflecting limited awareness of these aspects of digital health services. The awareness of clinical and core healthcare services was moderate (Mean = 58.49%, SD = 35.11), suggesting an intermediate level of awareness among participants.

Overall, the mean total digital health awareness score was 51.35% (SD = 30.69). Based on the predefined awareness categories, nearly half of the participants demonstrated low digital health awareness (*n* = 178, 45.5%), while 29.7% (*n* = 116) had moderate awareness and 24.8% (*n* = 97) had high awareness. These findings indicate that, despite the overall mean score falling within the moderate range, low digital health awareness was the most common category among participants.

Table 4 presents participants’ experience with digital health technologies and their patterns of digital health utilization. The findings revealed that more than two-thirds of the participants used the internet to search for health-related information (68.5%), whereas 31.5% reported not using the internet for health information seeking.

**Table 4:** Experience and digital health utilization patterns (*n* = 391).

Variable	No.	%
Use of the internet for health information seeking		
No	123	31.5
Yes	268	68.5
Digital health applications used by participants		
No applications used	45	11.5
Used NGHA app	110	28.1
Used Sehhaty app	152	38.9
Used both apps	77	19.7
Other apps are used	7	1.8

Regarding the use of digital health applications, most participants reported using at least one digital health application. The Sehhaty application was the most frequently used (38.9%), followed by the NGHA application (28.1%), while 19.7% of participants reported using both applications. Only 11.5% indicated that they did not use any digital health applications.

Overall, these findings suggest a relatively high level of engagement with digital health technologies among participants, particularly for health information seeking and the use of national digital health applications.

Table 5 illustrates the patterns of digital health service utilization across different applications among study participants.

**Table 5:** Patterns of digital health service utilization across applications.

Digital health service utilization pattern	Not used		NGHA app		Sehati app		Both applications	
	No.	%	No.	%	No.	%	No.	%
Navigation and access services								
Booking appointments through the application	69	17.6	144	36.8	136	34.8	42	10.7
Accessing laboratory and imaging results through the application	145	37.1	119	30.4	107	27.4	20	5.1
Viewing previous appointments and medical reports	134	34.3	112	28.6	119	30.4	26	6.6
Viewing prescribed medications through the application	126	32.2	121	30.9	117	29.9	27	6.9
Communication and consultation services								
Requesting medical consultations through the application	167	42.7	70	17.9	128	32.7	26	6.6
Accessing family medical team information	252	64.5	35	9.0	96	24.6	8	2.0
Medication and health management services								
Setting medication reminders	286	73.1	44	11.3	56	14.3	5	1.3
Requesting medication refills	153	39.1	113	28.9	111	28.4	14	3.6
Updating personal health information (weight, vital signs)	270	69.1	31	7.9	81	20.7	9	2.3
Viewing visit summaries and medical reports	259	66.2	50	12.8	68	17.4	14	3.6
Viewing and updating vaccination history	236	60.4	46	11.8	101	25.8	8	2.0
Administrative and family health service								
Managing family members’ health records	264	67.5	41	10.5	73	18.7	13	3.3
Sharing medical leave reports	181	46.3	78	19.9	109	27.9	23	5.9
Requesting emergency services	280	71.6	28	7.2	71	18.2	12	3.1
Digital support and integration services								
Viewing insurance cards	290	74.2	27	6.9	68	17.4	6	1.5
Linking the application with other health applications	272	69.6	28	7.2	84	21.5	7	1.8

The findings demonstrated that utilization varied considerably across service categories. Within the navigation and access services domain, booking appointments through digital health applications was among the most commonly utilized services, with utilization distributed across both the NGHA and Sehhaty applications. Similarly, moderate levels of utilization were observed for accessing laboratory and imaging results, viewing previous appointments, and reviewing prescribed medications.

In the communication and consultation services domain, requesting medical consultations showed moderate utilization, whereas accessing family medical team information was less frequently utilized, with a substantial proportion of participants reporting non-use.

Lower utilization rates were observed in the medication and health management services domain. Services such as medication reminders, updating personal health information, and viewing visit summaries demonstrated high levels of non-use among participants.

Similarly, administrative and family health services, including managing family members’ records and requesting emergency services, showed limited utilization across applications. Digital support and integration services represented the least utilized category, as most participants reported not using features related to viewing insurance cards or linking applications with other health applications.

Overall, the results indicate that participants primarily utilized digital health applications for basic healthcare access and appointment-related services, whereas more advanced, supportive, and integrative digital health features remained underutilized. These findings highlight variability in the extent of digital health engagement among patients with chronic illnesses.

Table 6 presents the mean percentage scores of digital health attitudes across two domains, in addition to the overall attitude score among participants.

**Table 6:** Mean percentage scores of digital health attitudes across domains.

Digital health attitude domain	Mean (%)	SD
Perceived trust and information value total	68.2	20.1
Behavioral impact and engagement attitude total	69.4	24.3
^a^Attitude total scoring	68.7	21.5
Attitude categories	No.	%
Negative attitude (<50%)	80	20.5
Moderate attitude (50–<75%)	113	28.9
Positive attitude (≥75%)	198	50.6

a Domain scores were calculated independently as percentage scores based on the maximum possible score for each domain; therefore, the domain percentages are not additive and do not sum to the overall attitude percentage score.

The findings showed that the overall digital health attitude score percentage was 68.7% (SD = 21.5), indicating generally positive attitudes toward digital health technologies. Domain-specific scores were comparable, with mean percentage scores of 68.2% for perceived trust and information value and 69.4% for behavioral impact and engagement attitude.

In terms of attitude categories, more than half of the participants demonstrated a positive attitude toward digital health applications (*n* = 198, 50.6%). Meanwhile, 28.9% (*n* = 113) of participants exhibited a moderate attitude, while 20.5% (*n* = 80) showed a negative attitude. Overall, positive attitudes were more prevalent than negative ones, indicating a generally favorable perception of digital health technologies among patients with chronic illnesses.

Table 7 demonstrates the correlation between digital health awareness and attitude scores among participants. The findings revealed a **strong, positive, statistically significant correlation** between digital health awareness and attitude scores (r=0.693,p<0.001). This indicates that participants with higher levels of digital health awareness were more likely to demonstrate positive attitudes toward digital health applications. The strength of the correlation suggests a substantial relationship between awareness and attitudes toward digital health technologies among patients with chronic diseases. These findings support the assumption that increasing awareness of digital health services may contribute to improving patients’ perceptions and acceptance of digital health applications.

**Table 7:** Correlation between digital health awareness and attitude scores (*n* = 391).

Variables	Digital health awareness total score		Attitude total score	
	*R*	*P*	*r*	*P*
1. Digital health awareness total score	1		0.693^a^	<0.001
2. Attitude total score	0.693^a^	<0.001	1	

*r*, Pearson correlation; *P*, *P* value of Pearson correlation.

*r* < 0.2: no correlation. *r*: 0.2-0.4: weak correlation. *r*: 0.4-0.6: A moderate correlation. *r*: 0.6-0.8: A strong correlation. *r* > 0.8: A perfect correlation.

a Correlation is significant at the 0.01 level (2-tailed).

Multiple linear regression analysis was conducted to identify predictors of attitudes toward digital health applications among patients with chronic diseases (Table 8). The multiple linear regression model was statistically significant (*F* = 142.166, *p* < 0.001), explaining 59.6% of the variance in attitude scores (*R*^2^ = 0.596; adjusted *R*^2^ = 0.591), indicating a strong model fit. Regression diagnostics indicated no evidence of multicollinearity, with tolerance values ranging from 0.628 to 0.714 and variance inflation factor (VIF) values ranging from 1.401 to 1.594. The Durbin–Watson statistic was 1.705, indicating acceptable independence of residuals.

**Table 8:** Multiple linear regression analysis predicting attitudes toward digital health applications among patients with chronic diseases (*n* = 391).

Predictor Variables	B	SE	*β*	T	*p*-value	95% CI	Tolerance	VIF
Constant	36.60	3.05	–	11.998	<0.001	30.60, 42.59	–	–
Age	−0.129	0.041	−0.120	−3.116	0.002	−0.211, −0.048	0.709	1.410
Use of the Internet for health information seeking	5.324	1.442	0.144	3.691	<0.001	2.488, 8.160	0.692	1.446
Digital health application use	5.036	0.683	0.283	7.378	<0.001	3.694, 6.378	0.714	1.401
Digital health awareness score	0.641	0.060	0.434	10.613	<0.001	0.522, 0.759	0.628	1.594
Model fit statistics	*R* = 0.772 *R*^2^ = 0.596 Adjusted *R*^2^ = 0.591 *F* = 142.166 *p* < 0.001 Durbin–Watson = 1.705							

Dependent variable: Total attitude score toward digital health applications.

B, unstandardized coefficient; SE, standard error; β, standardized coefficient; CI, confidence interval; VIF, variance inflation factor. Tolerance, tolerance statistic; Durbin–Watson assesses the independence of residuals.

Digital health awareness emerged as the strongest predictor of attitudes toward digital health applications (*β* = 0.434, *p* < 0.001), suggesting that higher awareness is associated with more positive attitudes. The use of digital health applications was also significantly associated with attitudes (*β* = 0.283, *p* < 0.001), indicating that participants who actively use digital health applications tended to have more favorable attitudes. In addition, the use of the internet for health information seeking was significantly associated with positive attitudes (*β* = 0.144, *p* < 0.001). Age showed a significant negative association with attitude scores (*β* = −0.120, *p* = 0.002), indicating that older participants tended to report less positive attitudes toward digital health applications. Overall, the findings suggest that digital health awareness and prior engagement with digital technologies play important roles in shaping positive attitudes toward digital health applications.

## Discussion

This study assessed awareness, attitudes, and experiences of using digital health services among patients with chronic diseases at King Abdulaziz Medical City. The findings provide important insights into how patients engage with digital health technologies in a tertiary care setting and highlight key determinants influencing acceptance and utilization. Overall, the results demonstrated that nearly half of the participants had low digital health awareness, alongside selective but functionally oriented utilization patterns and generally positive attitudes toward digital health services. Digital health awareness emerged as the strongest predictor of positive attitudes, highlighting its important role in shaping patients’ engagement with digital health technologies. These findings suggest that digital health adoption among patients with chronic diseases is still transitioning from basic exposure toward more meaningful and sustained engagement, rather than full integration into routine self-management.

The sociodemographic profile revealed a predominantly middle-aged, married population with diverse educational and occupational backgrounds, which is consistent with the typical epidemiological distribution of chronic diseases. From a critical interpretive perspective, this demographic composition is highly relevant because engagement with digital health services is not uniform but strongly shaped by age, education, and digital literacy levels. Prior Saudi evidence has shown that variability in education and digital competence significantly affects readiness to adopt and effectively use digital health technologies (23). In addition, the high prevalence of multimorbidity, particularly diabetes and hypertension, reflects a well-established regional burden of chronic disease. However, multimorbidity introduces greater complexity in self-management and care coordination, which may limit patients’ ability to fully benefit from digital health platforms, especially when systems are fragmented or not sufficiently user-centered (24). This underscores the need for simplified, integrated, and patient-centered digital health solutions tailored to complex chronic care needs.

The study demonstrated that nearly half of the participants had low digital health awareness, although awareness varied across different domains. Participants showed greater recognition of the availability of digital health services than of advanced digital health functionalities, indicating that awareness remained largely superficial rather than operational. In other words, while many participants were aware of the existence of digital health services, they lacked the functional understanding required for meaningful engagement in chronic disease management. This finding aligns with previous Saudi research showing that digital health awareness is often restricted to basic recognition of digital platforms rather than deeper engagement with their full capabilities (25). From a theoretical perspective, awareness represents an important early stage in technology acceptance models such as UTAUT, where it influences perceived usefulness and behavioral intention (26). The present findings suggest that many patients remain at this early stage of technology adoption, highlighting the need for stronger patient education and greater integration of digital health support within routine clinical care.

In terms of utilization, participants primarily used digital health applications for basic administrative functions such as appointment booking, laboratory results access, and prescription viewing. This pattern reflects a task-oriented rather than clinically integrated form of digital engagement. Similar findings have been reported in global literature, where patients tend to adopt digital health technologies primarily to reduce administrative burden while avoiding more complex self-management features (27, 28). Despite significant advancements in digital health infrastructure, real-world usage often remains limited to low-complexity functions due to usability constraints and the limited perceived value of advanced features. In the Saudi context, comparable utilization patterns have been observed in platforms such as Sehhaty, where engagement remains concentrated on administrative services rather than behavioral health management tools (29, 30). This suggests that although digital infrastructure is well developed, patient engagement remains shallow, reflecting what can be conceptualized as a “digital utilization ceiling,” where users stop at basic functions due to insufficient training, limited reinforcement by healthcare providers, and unclear perceived benefits of advanced features. These findings have important implications for both platform design and patient education. Simplifying advanced digital health features, improving user interface navigation, and providing user-centered educational support may facilitate broader adoption of functionalities that directly support chronic disease self-management, such as medication management, health record access, and remote monitoring. Greater involvement of healthcare providers in introducing and reinforcing these features during routine clinical encounters may further enhance patients’ confidence and sustained engagement with digital health services.

The study also found generally positive attitudes toward digital health services, with more than half of participants expressing favorable perceptions. This aligns with global trends indicating increasing acceptance of digital health technologies following the expansion of telemedicine services, particularly after the COVID-19 pandemic (31, 32). However, the presence of neutral and negative attitudes indicates that acceptance is not uniform across all patients. Variability in trust, usability perceptions, and engagement suggests persistent concerns regarding data privacy, system reliability, and ease of use. Prior studies have emphasized that trust is a central determinant of sustained digital health adoption, particularly among patients with chronic diseases who require continuous interaction with healthcare systems (25). Importantly, positive attitudes alone are insufficient to ensure behavioral adoption, highlighting the persistent intention–behavior gap frequently reported in digital health literature.

A strong positive correlation between digital health awareness and attitudes was observed, indicating that higher awareness is associated with more favorable perceptions of digital health services. This finding is consistent with UTAUT-based theoretical frameworks, which suggest that knowledge and familiarity enhance perceived usefulness and acceptance of technology (26). Empirical evidence from Saudi digital health studies also supports this relationship, showing that awareness improves trust, satisfaction, and willingness to use digital health platforms (23). This indicates that awareness is not merely informational; it also functions as a key behavioral determinant shaping how patients evaluate and engage with digital health systems.

Regression analysis further confirmed that digital health awareness, application use, and internet-based health information seeking were significant predictors of positive attitudes, while age showed a negative association. Digital health awareness emerged as the strongest predictor, reinforcing its central role in shaping acceptance and engagement. The regression model explained 59.6% of the variance in attitudes toward digital health applications, indicating that digital health awareness and digital engagement behaviors explained a substantial proportion of the variation in patients’ attitudes toward digital health technologies. Nevertheless, approximately 40% of the variance remained unexplained, suggesting that additional factors, such as socioeconomic characteristics, health status, previous experience with technology, and healthcare system factors, may also influence patients’ attitudes and should be explored in future research. This is consistent with recent evidence indicating that awareness strongly influences mHealth adoption and user satisfaction (25, 33). Prior experience with digital applications also contributed significantly to positive attitudes, suggesting that familiarity reduces uncertainty and enhances confidence through experiential learning processes (29). In addition, individuals who actively seek health information online demonstrated more positive attitudes, reflecting broader digital engagement behavior associated with higher digital readiness (34). The negative association with age aligns with established literature indicating that older adults may face barriers related to digital literacy and usability challenges; however, these barriers should not be interpreted as fixed and can be mitigated through targeted education, simplified interface design, and continuous support (24, 35).

From a broader systems perspective, the findings highlight a persistent gap between the availability of digital health services and their meaningful utilization. As Topol (2019) and Alharbi & Jahan (2023) (27, 36) emphasize, digital transformation in healthcare requires not only technological advancement but also human-centered design that aligns with patient needs, capabilities, and real-world workflows. In the Saudi healthcare context, where digital transformation is rapidly advancing, the present findings suggest that success depends not only on infrastructure development but also on strengthening patient awareness, improving usability, and embedding digital health literacy within routine clinical practice (23).

Because this study employed a cross-sectional design, the observed associations should be interpreted as relationships rather than evidence of causality.

This study demonstrates that nearly half of the patients with chronic diseases had low digital health awareness, while utilization of digital health services remained selective and attitudes toward digital health applications were generally positive. Digital health awareness emerged as the strongest predictor of positive attitudes, underscoring its central role in shaping patients’ engagement with digital health technologies. Overall, the findings suggest that digital health implementation in chronic care remains predominantly system-driven with limited patient empowerment, reflecting a transitional rather than fully integrated stage of digital transformation. These findings highlight the need for targeted educational interventions, improved system usability, and stronger integration of digital health tools into routine clinical care to enhance patient engagement and maximize the benefits of digital health technologies for chronic disease management.

## Limitations

This study has several limitations that should be considered when interpreting the findings. First, the cross-sectional design limits the ability to establish causal relationships between digital health awareness, attitudes, and utilization patterns. Although significant associations were identified, temporal sequencing cannot be confirmed. Second, the study was conducted at a single tertiary healthcare facility (King Abdulaziz Medical City, Jeddah), and the characteristics of patients receiving care at this institution may differ from those of patients in other healthcare settings across Saudi Arabia. Therefore, the findings should be interpreted with caution and should not be generalized to all patients with chronic diseases in the country. Third, the use of non-probability convenience sampling may have introduced selection bias, as patients who were more interested in or familiar with digital health technologies may have been more willing to participate, potentially influencing the observed levels of awareness, attitudes, and utilization. Fourth, reliance on self-reported data may have introduced response bias, including social desirability and recall bias, particularly in reporting digital health utilization behaviors. Fifth, the study did not include a qualitative component, which could have provided deeper insight into the reasons underlying the limited use of advanced digital health features. Finally, differences in participants’ digital literacy levels may have influenced their interpretation of questionnaire items, potentially affecting the accuracy of their responses.

## Implications of the study

### Implications for practice

The findings of this study have important implications for healthcare practice and digital health implementation. The finding that nearly half of the participants demonstrated low digital health awareness, together with the selective utilization of digital health services, suggests that the availability of digital health services alone is insufficient to ensure meaningful engagement. Healthcare providers should play an active role in enhancing patients’ digital health awareness through structured education during clinical encounters, including brief demonstrations of digital health applications and their functions beyond basic administrative use. Greater emphasis should also be placed on promoting the use of advanced digital health features, such as medication management, health record access, and remote health monitoring, to maximize the benefits of digital health technologies in chronic disease management.

In addition, the identification of awareness as the strongest predictor of attitudes highlights the importance of integrating patient activation strategies into routine care. Hospitals and healthcare systems should consider embedding digital health onboarding processes within outpatient services to support patient onboarding and sustained engagement. Furthermore, improving the usability and simplicity of digital health platforms is essential, particularly for patients with multimorbidity and older adults, to reduce usability barriers and enhance adoption.

### Implications for future research

Future research should build on these findings by employing longitudinal designs to better understand how awareness and attitudes evolve and how they influence sustained digital health use. Qualitative studies are also recommended to explore in depth the barriers and facilitators influencing the underutilization of advanced digital health features, particularly among patients with chronic diseases.

Additionally, comparative studies across different healthcare settings and regions within Saudi Arabia are needed to assess variability in digital health engagement. Future research should also examine the role of digital literacy interventions and training programs in improving meaningful use of digital health services. Integrating behavioral theories with the implementation of science frameworks may further enhance understanding of adoption dynamics in chronic disease populations.

### Implications for education

The findings highlight the need to integrate digital health literacy into patient education programs as well as healthcare professional training. For patients, structured educational interventions should focus not only on awareness but also on practical skills required to use advanced digital health features, such as medication management, health tracking, and communication tools.

For healthcare professionals, training programs should emphasize the importance of patient digital engagement and equip clinicians with the skills to guide patients in using digital health platforms effectively. Incorporating digital health competencies into medical, nursing, and allied health curricula is essential to ensure that future healthcare providers are prepared to support digital transformation in clinical practice.

## Conclusion

This study provides valuable insights into digital health awareness, attitudes, and utilization among patients with chronic diseases attending King Abdulaziz Medical City, Jeddah. Nearly half of the participants demonstrated low digital health awareness, while attitudes toward digital health applications were generally positive. However, utilization remained largely limited to basic administrative functions, indicating a clear gap between the availability of digital health services and meaningful engagement with advanced functionalities.

The core contribution of this study is the identification of this awareness–utilization gap and the finding that digital health awareness was the strongest predictor of attitudes toward digital health applications. The regression model explained 59.6% of the variance in attitude scores, highlighting the important role of awareness and digital engagement in shaping patients’ perceptions of digital health technologies. These findings extend the understanding of digital health adoption among patients with chronic diseases in the Saudi healthcare context by emphasizing that digital transformation requires more than infrastructure availability.

Overall, the findings highlight the need for targeted patient education, improved system usability, and stronger integration of digital health services into routine clinical workflows. Moving beyond basic administrative use toward meaningful engagement with advanced digital health functionalities may help maximize the benefits of digital technologies for chronic disease management and support more patient-centered digital health transformation in similar tertiary healthcare settings.

## Data Availability

The raw data supporting the conclusions of this article will be made available by the authors, without undue reservation.
